# Hygiene and Nursing in Belgium

**Published:** 1923-04

**Authors:** 


### HYGIENE AND NURSING IN BELGIUM.

A NEW Institute of Hygiene is shortly to be created in Antwerp, and a committee has been formed of Belgian architects with a view to designing and erecting the necessary buildings. The site selected has a surface of more than two acres and the Institute will include a bacteriological laboratory, comprising laboratories for the provincial analysis service, for the preparation and sterilisation of cultures, a chemical laboratory, a micrographic installation, incinerating furnaces, &c. A clinic, with operating theatres and anæsthetising and sterilising annexes, an X-ray department, two wards of 10 beds each, two dispensaries and a laboratory for the study of tropical, social and professional diseases, will be annexed to the Institute. There will also be a hall for lectures and classes accessible to the public, one or two rooms for the installation of a museum of hygiene, two meeting-halls for the use of medical societies and official organisations attached to the Institute, and a clubroom adjoining the library.

### The Organisation of Nurses.

It is proposed to create in Belgium a corps of trained nurses who would have, in the same way as doctors and chemists, a certain official standing. All nurses will either be attached to a service of medicine or surgery, to a special service for diseases of the nose, throat and ear, to an anti-tubercular dispensary, to a sanatorium, or to a maternity hospital, or they will be visiting nurses. In addition to all this, nurses will have subordinates to direct and teach, until the time when they themselves become professors in the training-schools for nurses. The whole question has given rise to much discussion in Belgium, and is as yet far from being settled. Several different proposals have been put forward as to the length of time to be covered by the course of training. Three years are considered necessary by some authorities, two by others, while a third suggestion is that three categories should be formed—nurses of the first degree taking a six months course of training, nurses of the second degree studying for 18 months, and nurses of the third degree doing the full training of about three years.

### A GREAT HOSPITAL EXTENSION.

THE committee of the Carnarvonshire and Anglesey Infirmary at Bangor is engaged in raising the balance of the £20,000 required to complete an important programme. The scheme is to increase the beds from 52 to 120 ; to provide a new theatre, an enlarged X-ray room, and special departments ; and to enlarge and improve the nurses' home, the boiler house and the laundry. A small number of wards for paying patients will be included and four beds reserved for special clinics in connection with the public health services. To gain the conditional grant of £8,000 from the Red Cross, £2,500 is required, and to complete the programme £6,000. When all this has been done, the institution will be able to claim that it is a fully-equipped general hospital ; men, women and children will be provided for ; and it is, therefore, to be hoped that the public will support the committee.

